# Prolactin Monitoring in Antipsychotic Prescribing: A Trust-Wide Completion of Audit Cycle

**DOI:** 10.1192/bjo.2026.11676

**Published:** 2026-06-30

**Authors:** Amy (Hyemin) Kim, Azhar Emambocus, Thomas Wiggins, Seunghyeon Moon, Timothy Hammond

**Affiliations:** SABP, Guildford, United Kingdom

## Abstract

**Aims:**

Our aim is to evaluate the trust-wide adherence to NICE and Maudsley guidelines for baseline and follow-up prolactin monitoring in patients prescribed medium to high risk antipsychotic medications across inpatient and outpatient services of Surrey and Borders Partnership (SABP) NHS Foundation Trust. This re-audit was performed to complete the cycle by assessing the impact of trust-wide recommendations implemented following the initial 2021 SABP audit on prolactin monitoring.

**Methods:**

Data were collected for inpatients admitted to working- and old-age wards across the trust during September 2025 (n=160), including those prescribed antipsychotics with medium to high risk of hyperprolactinaemia. Patients were categorised according to whetherthey were established on an antipsychotic prior to admission or initiated on a new antipsychotic during admission.

For patients already prescribed antipsychotics, electronic records were reviewed to determine whether serum prolactin levels had been measured within the preceding 12 months, including tests performed in community settings or during previous admissions. For patients commenced on a new antipsychotic during admission, compliance with baseline prolactin measurement prior to treatment initiation was assessed. Where elevated prolactin levels were identified, further data were collected to determine whether symptoms of hyperprolactinaemia had been assessed, whether appropriate management had been initiated, and whether repeat prolactin testing had been performed in line with guidance. Data were obtained retrospectively from electronic care records, SystmOne, and the ICE pathology system.

**Results:**

During September 2025, compliance with serum prolactin monitoring for patients prescribed medium- to high-risk antipsychotics was 100% both prior to admission and during inpatient initiation. This represents a substantial improvement from compliance rates of 47% and 43%, respectively, identified in the 2021 audit.

Of the 25 inpatients commenced on medium- to high-risk antipsychotics who had prolactin levels measured, 9 were found to have hyperprolactinaemia. Appropriate management was initiated in 4 of these cases, and repeat prolactin testing was performed in 3. Compared with the 2021 audit, this reflects an 11% improvement in appropriate management, alongside a 33% reduction in repeat prolactin testing.

**Conclusion:**

This re-audit demonstrates a marked improvement in compliance with recommended prolactin monitoring, achieving full adherence to guidelines across inpatient services. However, despite improved detection, there remains a significant gap in the subsequent management of hyperprolactinaemia. These findings highlight the need for further quality improvement initiatives, including the introduction of a standardised management pathway, supported by electronic prompts and targeted clinician education, to ensure timely and consistent management of abnormal prolactin results.

